# The relationship between Alzheimer’s disease and intestinal microflora structure and inflammatory factors

**DOI:** 10.3389/fnagi.2022.972982

**Published:** 2022-11-09

**Authors:** Su-shan Wang, Xiao-hui Li, Ping Liu, Jing Li, Li Liu

**Affiliations:** ^1^Department of General Practice, The Sixth Medical Center of PLA General Hospital, Beijing, China; ^2^Six Health Care Department, The Sixth Medical Center of PLA General Hospital, Beijing, China

**Keywords:** Alzheimer’s disease, intestinal microflora, inflammatory factors, TNF-α, IL-6

## Abstract

To analyze the structural characteristics of intestinal microflora and changes of serum inflammatory factors of the Alzheimer’s disease, and to explore the relationship between them and dementia, we selected 30 patients in the AD group and 30 patients in the normal group, and collected stool samples to analyze the intestinal flora structure characteristics of the two groups of patients, and statistically analyzed the inflammatory cytokines TNF-α, IL-1β, IL-6, and IL-8 by ELISA from the venous blood of the two groups. The results show that the dominant *Bacteroides* in the two groups are *Bacteroides*, *Firmicutes*, *Proteobacteria*, and *Actinobacteria*. The abundance of *Bacteroides*, *Firmicutes*, and *Proteobacteria* in the AD group shows a statistical difference. At the genus level, the abundance of anti-inflammatory bacteria such as *Lactobacillus*, *Bifidobacterium*, and *Ruminococcus* drops in AD group, while the abundance of pro-inflammatory bacteria such as *Escherichia* and *Enterococcus* raises. Statistical analysis of inflammatory cytokines in the two groups suggests that TNF-α and IL-6 levels significantly increase in the AD group, with statistical differences. Therefore, it is speculated that the increased abundance of pro-inflammatory bacteria in intestinal flora may lead to or aggravate neuroinflammation through the release of inflammatory factors, thus further leading to the occurrence and development of AD.

## Introduction

With the aging process of society, the incidence of dementia is increasing year by year and has become a common disease of the elderly. It is reported that by 2015, the number of global dementia patients was about 47 million and is expected to reach 131 million by 2050 (Livingston et al., 2017; Watermeyer and Calia, 2019), which is bound to place a heavy burden on families and society. Alzheimer’s disease dementia accounts for 60∼80%, which is the leading cause of disability and death in the elderly (Alzheimer’s Association, 2019; Alzheimer’s Disease International, 2019).

Alzheimer’s disease (AD) is a chronic neurodegenerative disease common to the elderly, characterized by a gradual decline in cognitive function that eventually leads to loss of memory, thinking, and reasoning capability. The pathological characteristics of AD are amyloid protein (Aβ) deposition and neurofibrillary tangles (NFT). However, the specific pathogenesis of AD is still unclear. It is reported that environmental factors and genetic factors play a role in developing AD. Many previous studies (Zhao and Gong, 2015; Swerdlow, 2018; Nguyen et al., 2020) suggest that mitochondrial dysfunction, insulin resistance and cerebral hypoperfusion may mediate, drive or even trigger pathological molecular cascades in AD, ultimately promoting Aβ accumulation, tau hyperphosphorylation, synaptic degeneration, and neuronal dysfunction. However, these studies cannot fully explain AD’s etiology and potential pathophysiological mechanism.

In recent years, studies have shown that intestinal flora is related to the incidence and progression of AD. The Human gut microbiome is considered as a “second brain” (Schneider et al., 2019; Sochocka et al., 2019). Some essential vitamins and substances involved in developing the central nervous system and immune regulation are produced by intestinal microbiota (Picca et al., 2018). In addition to destroying the stability of the intestinal environment, the imbalance of intestinal microorganisms will also affect behavior, learning, and memory, as well as the occurrence of nervous system diseases (Luczynski et al., 2016; Tremlett et al., 2017). Previous studies have suggested that the mechanism of intestinal microbiota leading to AD may start from the imbalance of intestinal microbiota, the development of local and systemic inflammation, and the disorder of the enteric-brain axis. The increased permeability of the intestinal epithelial barrier leads to the invasion of bacteria, viruses, and their neuroactive products and leads to neuroinflammatory responses in the brain, leading to AD (Sochocka et al., 2019). Combined with previous researches, we suggest that the occurrence of AD may be related to the low level of the inflammatory response caused by the structural disorder of intestinal flora. The change of intestinal flora structure and the increase of intestinal flora with pro-inflammatory effects, such as *Bifidobacterium*, will lead to the increase of inflammatory factor level, further lead to neuroinflammation and promote the occurrence and development of AD. This study aims to observe the structural characteristics of intestinal flora and the changes of serum inflammatory factors in patients with Alzheimer’s disease and explore the relationship between them and Alzheimer’s disease.

## Data and methods

### General information

We routinely performed preliminary screening of MMSE + MOCA on patients in the neurology outpatient department and ward of the cadres of our hospital. Patients with cognitive dysfunction were judged according to the diagnostic criteria of AD stipulated by IWG-2 (2014). Those meeting the diagnostic criteria of AD were enrolled in the AD group, and those without both cognitive dysfunction and AD dementia were classified as the normal group. In addition, all the patients in the above two groups had no infectious diseases, gastrointestinal diseases, or oral probiotics within the last 3°months after screening. The cut off for the subdivision of the sample between AD group and normal group is as follows:

Normal Group: MMSE ≥ 27 MOCA ≥ 26

Alzheimer’s disease Group: secondary school education and above: MMSE ≤ 24

primary school education: MMSE ≤ 20

illiterate: MMSE ≤ 17

MOCA:11 ∼ 21

### Inspection methods

Three°ml of fasting venous blood was taken from the two groups early morning and centrifuged at 2,000r/min for 10°min to separate the upper serum. Inflammatory cytokines TNF-α, IL-1β, IL-6, and IL-8 were detected by ELISA.

Collect fresh, middle, rear, and internal fecal samples from the two groups. Each sample needs 2G. After collection, it shall be frozen and stored in the −80° refrigerator immediately. Remember to freeze and repeatedly thaw during storage.

Stool DNA Kit (Omega, USA) extracted total DNA from fecal samples. The specific operation was carried out according to the instructions. The extracted whole-genome DNA was inspected, and the qualified bacteria were amplified in the V4 variable region of 16S rDNA. The amplicons were recovered, purified, and quantified, and double-ended sequencing was performed according to the standard process of the Illumina miseq 250 sequencing platform. The double-ended sequences obtained by sequencing were spliced into a target region sequence, and the target sequence was filtered by quality control. The filtered sequence is compared with the reference database, and the chimeric sequence is removed to obtain the final optimized sequence. Out cluster analysis and species taxonomic annotation were carried out based on the optimized sequence.

### Statistical analysis

SPSS statistical software was used to analyze the data. The measurement was expressed as (x ± s). One way, an OVA was used when the data were in line with normal distribution and the variance was uniform; otherwise, the rank-sum test was used; the Chi-square test was used to compare the rates. *P* < 0.05 was considered statistically significant.

## Results

### Basic characteristics

Thirty subjects were included in the AD group and the normal group respectively. Statistical analysis results showed that there were no significant statistical differences in age gender, smoking history, drinking history and general diet between the two groups (Table 1). (In this study, the subjects were randomly selected from the inpatients in the cadre ward, so the proportion of males was higher than females).

**TABLE 1:** Basic characteristics of the subjects in each group.

	AD group (*n* = 30)	Normal group (*n* = 30)	*P-value*
Age in years, mean ± SEM	79.80 ± 8.55	78.23 ± 9.71	0.510
Gender			0.542
Male	24 (80.0%)	22 (73.3%)	
Female	6 (20.0%)	8 (26.7%)	
Smoking			0.432
Yes	14 (46.7%)	11 (36.7%)	
No	16 (53.3%)	19 (63.3%)	
Drinking			0.795
Yes	12 (40.0%)	10 (33.3%)	
No	18 (60.0%)	20 (66.7%)	
Diet			1.000
Meat based diet	2 (6.7%)	1 (3.3%)	
Vegetarian diet	1 (3.3%)	1 (3.3%)	
Omnivorous diet	27 (90.0%)	28 (93.3%)	

### Coverage of sample flora

The Coverage index refers to the sequencing coverage of each sample strain. Each ‘group’s values are above 0.9, indicating a low probability of not being measured in the sample sequence (Table 2).

**TABLE 2:** Sequencing coverage rate of each group.

	Coverage index
AD group	0.9965 ± 0.00119
Normal group	0.9966 ± 0.00142

### Alpha diversity analysis

Alpha diversity analysis reflects the richness of microorganisms in samples by diversity index. We selected the Shannon index and Simpson index for statistical analysis. The larger Shannon index and the smaller Simpson index, the higher species diversity in the sample is (Table 3).

**TABLE 3:** Alpha diversity index of each group.

Diversity	AD group	Normal group	*P-value*
Shannon	2.740 ± 0.222	3.537 ± 0.206	0.000*
Simpson	0.097 ± 0.021	0.091 ± 0.019	0.348

*Means that the difference is considered statistically significant (*P* < 0.05).

### The structure and abundance of bacteria at the phylum level in each group

**FIGURE 1:**
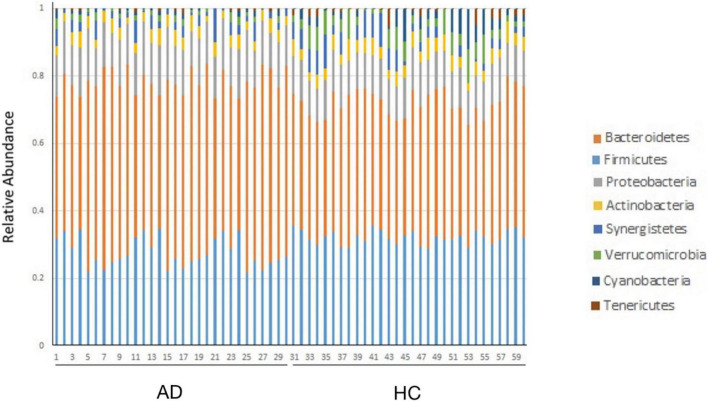
The bacterial community structure of the two groups of samples at the phylum level.

The bacterial community structure of the two groups of samples at the phylum level is shown in Figure 1. The dominant phyla are *Bacteroidetes*, *Firmicutes*, *Proteobacteria*, and *Actinomycetes*, respectively. Compared with the normal, the abundance of *Bacteroidetes* (*P* = 0.014) and *Proteobacteria* (*P* = 0.004) in the AD group increased, and the abundance of *Firmicutes* decreased (*P* = 0.000) (Table 4).

**TABLE 4:** The structure and abundance of bacteria at the phylum level in each group.

	AD group	Normal group	*P-value*
*Bacteroidetes*	0.524 ± 0.098	0.402 ± 0.037	0.014*
*Firmicutes*	0.276 ± 0.043	0.321 ± 0.021	0.000*
*Proteobacteria*	0.131 ± 0.017	0.112 ± 0.011	0.004*
*Actinobacteria*	0.034 ± 0.010	0.040 ± 0.014	0.077

*Means that the difference is considered statistically significant (*P* < 0.05).

### The inflammatory factors and abundance of anti-inflammatory bacteria and pro-inflammatory bacteria in each group

The levels of inflammatory factors TNF-α (*P* = 0.004) and IL-6 (*P* = 0.001) are significantly different between the normal group and AD group, but there are no significant differences in the levels of IL-1β (*P* = 1.000) and IL-8 (*P* = 0.072) between the two groups.

We selected some of the dominant flora in the gut, such as *Bifidobacterium*, *Lactobacillus*, *Ruminococcus* with anti-inflammatory effects and *Escherichia*, *Klebsiella*, and *Enterococcus* with pro-inflammatory effects, and statistically analyzed the abundance values of the above bacteria in the two groups of samples. Compared with the normal, the abundance of *Ruminococcus* (*P* = 0.000), *Lactobacillus* (*P* = 0.000), and *Bifidobacterium* (*P* = 0.000) in the AD group decreased, and the abundance of *Escherichia coli* (*P* = 0.000) and *Enterococcus* increased (*P* = 0.025) (Table 5).

**TABLE 5:** Inflammatory factors and abundance of anti-inflammatory bacteria and pro-inflammatory bacteria in each group.

		AD group	Normal group	*P-value*
Inflammatory factors	TNF-α (ug/ml)	11.46 ± 4.25	8.24 ± 3.38	0.004*
	IL-1 β (ug/ml)	11.37 ± 2.60	11.01 ± 2.35	1.000
	IL-6 (pg/ml)	7.81 ± 3.42	5.05 ± 1.93	0.001*
	IL-8 (ug/ml)	15.98 ± 4.38	13.42 ± 4.36	0.072
Abundance of bacteria	*Ruminococcus*	0.027 ± 0.015	0.042 ± 0.016	0.000*
	*Lactobacillus*	0.072 ± 0.026	0.111 ± 0.018	0.000*
	*Bifidobacterium*	0.002 ± 0.001	0.003 ± 0.002	0.000*
	*Enterococcus*	0.030 ± 0.011	0.019 ± 0.010	0.000*
	*Escherichia*	0.094 ± 0.203	0.013 ± 0.005	0.025*
	*Klebsiella*	0.020 ± 0.008	0.017 ± 0.008	0.551

*Means that the difference is considered statistically significant (*P* < 0.05).

## Discussion

Alzheimer’s disease is an age-related neurodegenerative disease, but its etiology and mechanism are not well-understood, and there is no effective treatment in the clinic. At present, the relevant drug treatment just can delay the progress of the disease to a certain extent. For a long time, the research on the pathogenesis of AD has never stopped. The relationship between intestinal flora, neuroinflammation, and AD occurrence and development is a hot topic.

### Intestinal flora

A large number of microorganisms settle in the human body. It is estimated that there are 500∼1,000 kinds of bacteria in the human body (Gilbert et al., 2018), most of which settle in the intestine (Ursell et al., 2012). Intestinal microorganisms play a vital role in the host’s health, such as defeating invading pathogens, producing neurotransmitters, regulating the immune system and so on. Recent studies have shown that the occurrence and development of neurodegenerative diseases such as AD are related to changes in intestinal flora (Al Bander et al., 2020). Intestinal flora regulates the development and homeostasis of the central nervous system through immune, circulatory and neural pathways. In turn, the central nervous system shapes the intestinal microbial community through stress and endocrine response. The two-way effect of this gut-brain axis may be related to complex central nervous system diseases (Tremlett et al., 2017). The mechanism of intestinal flora leading to AD may begin with the imbalance of intestinal microbiota, the development of local and systemic inflammation, and the imbalance of the intestinal brain axis. The increase of intestinal epithelial barrier permeability leads to the invasion of bacteria, viruses, and their neuroactive products and causes the neuroinflammatory response of the brain, leading to AD (Sochocka et al., 2019). Our study brings valuable information about the correlation between intestinal flora and AD by exploring intestinal flora’s diversity and structural changes in patients with AD and normal cognition.

In this study, we sequenced the feces of AD patients and normal elderly patients. The sequencing depth of the two groups of samples was more than 99%. Alpha diversity analysis showed that the diversity of flora in AD patients was significantly lower than that in the normal group, suggesting that AD may be related to the decline of flora diversity to a certain extent. The flora structure analysis showed significant differences in the abundance of *Bacteroidetes*, *Proteobacteria*, and *Firmicutes* between the AD and normal groups. Vogt (Vogt et al., 2017) found that *Firmicutes* decreased and *Bacteroidetes* increased in the intestinal microbiota of AD patients, which was consistent with the results of this study. However, some studies have shown that *Bacteroidetes* in the intestinal microbiota of AD patients are reduced (Zhuang et al., 2018). The small sample size of these studies makes it difficult to draw consistent conclusions, so studies with larger samples are needed to provide more reliable evidence. Studies have shown that the ratio of *Firmicutes* to *Bacteroidetes* gradually decreases with age, accompanied by immune system disorders, and low-level intestinal inflammatory response gradually increases (Odamaki et al., 2016). *Bacteroidetes* belong to Gram-negative bacteria and can secrete a large amount of lipopolysaccharide. Lipopolysaccharide may act on Toll-like receptor four in leukocytes and microglia and promote nuclear factors κ*B* mediated cytokine production, thereby, ncreasing Aβ Level, damaging oligodendrocytes and myelin sheath. At the same time, Aβ 1∼42 are also agonists of Toll-like receptor four, which can cause Aβ. The level increases, forming a vicious circle and aggravating AD’s progress (Zhan et al., 2018). *Proteobacteria*, which only account for a small part of the intestinal tract of healthy adults (Faith et al., 2013), once their abundance increases excessively, the ability of inherent internal bacteria to resist colonization by foreign pathogens will decrease, further promoting inflammatory response and pathogen invasion. Therefore, *Proteobacteria* enrichment is also considered to be a marker of intestinal flora disorder, with potential diagnostic value (Shin et al., 2015).

### Alzheimer’s disease, inflammation, and intestinal flora

Many recent studies have found that immune and inflammatory control regulation is one of the related processes involved in the pathogenesis of neurodegenerative diseases. A large number of studies have shown that the participation of neuroinflammation plays a crucial role in the process of neuropathological changes observed in AD (Finneran and Nash, 2019). The key players currently known to be responsible for the induction of neuroinflammation are activated microglia and astrocytes (Ahmad et al., 2019). Long-term chronic inflammation and excessive microglia stimulation can increase the neuroinflammatory signal transduction of proinflammatory cytokines, reactive oxidation and nitrous stress sources, resulting in the death of neurons and glial cells and further promoting the occurrence and development of AD (Li et al., 2016). The inflammatory cytokines released by microglia in the elderly brain are mainly IL-1β, IL-6, and TNF-α (Ritzel et al., 2015).

We compared the levels of the TNF-α, IL-1β, IL-6, and IL-8 which are four proinflammatory factors in two groups of subjects. Results found that TNF-α and IL-6 levels are significantly higher than those in the normal group, which further confirm that inflammatory factors are involved in the occurrence of AD, especially TNF-α, IL-6.

TNF-α is a proinflammatory cytokine produced mainly by macrophages and monocytes and is involved in normal inflammatory and immune responses. The evidence found from previous studies (Di Bona et al., 2009; Swardfager et al., 2010) indicates that TNF-α is related to the pathogenesis of AD pathology and TNF-α is the primary signal molecule affecting memory and cognitive function (Kim et al., 2017). Previous studies have shown that overexpression of TNF-α can stimulate microglia to express inflammatory factors, thus promoting the formation of Aβ plaques (Dal Pra et al., 2008), causing neurotoxic cascade reactions (Wyss-Coray and Mucke, 2002), competing with neuro-nutrients, and leading to neuronal aging (Roux et al., 2001). In this study, we found that the level of TNF-α in AD group was significantly higher than that in the normal group. Combined with previous studies, it was further confirmed that TNF-α was involved in the occurrence and development of AD.

As an important member of the cytokine network, IL-6 can regulate the growth and differentiation of various cells, as well as the immune response, and participate in the pathological process of various clinical diseases. At the same time, IL-6 can promote the proliferation of T cells and B cells and participate in the body’s inflammatory response. Analysis in this study showed that IL-6 levels in AD patients were significantly higher than those in the normal group. This is consistent with previous meta-analyses (Swardfager et al., 2010). However, some studies have found that the increase of peripheral blood IL-6 level precedes the onset of Alzheimer’s disease rather than during the onset of Alzheimer’s disease (Uslu et al., 2012).

IL-1β is produced by macrophages, endothelial cells, and astrocytes. Overexpression of IL-1β can impair microglia Aβ scavenging function (Wang et al., 2015) and induce blood-brain barrier disruption, thereby increasing Aβ deposition (Wang et al., 2014). On the contrary, studies have shown that IL-1β released by microglia has a protective effect on AD (Ng et al., 2018). IL-1β not only reduces A β production by enhancing α-lysis of APP, but also alleviates amyloid lesions by increasing clearance mechanisms. Overexpression of IL-1β can enhance microglial phagocytosis and lead to plaque clearance (Matousek et al., 2012). In summary, IL-1 β plays a complex role in AD, and its expression and mechanism in AD need to be further studied.

Normal brain tissue can produce a certain amount of IL-8. Brain-derived IL-8 is involved in normal brain tissue metabolism and function. Peripheral IL-8 is involved in immune inflammation. Neurological diseases such as AD can inhibit the production of brain-derived IL-8, while inflammatory cytokines such as IL-1β and TNF-α produced by inflammation caused by neurological diseases can further induce the production of IL-8 by endothelial cells and neutrophils, leading to inflammatory chemotaxis and thus aggravating the damage of this region. Thus, IL-8 has a dual role. In this study, there was no significant difference in IL-1β and IL-8 levels between the two groups, which may be due to the complex mechanism of IL-1β and IL-8 on the one hand, and the small sample size, which needs to be further expanded to further confirm the role of IL-1β and IL-8 in the occurrence and progression of AD.

At the bacterial level, we selected some of the dominant flora in the gut, such as *Bifidobacterium*, *Lactobacillus*, *Ruminococcusi* with anti-inflammatory effects and *Escherichia*, *Klebsiella*, and *Enterococcus* with pro-inflammatory effects. Studies have shown that *Escherichia* includes a considerable number of pathogenic bacteria, which can cause intestinal inflammatory reactions under certain conditions (Wall et al., 2014; Potgieter et al., 2015). In the past, *Enterococcus* was considered harmless to the human body. In recent years, studies have confirmed that some *Enterococcus* have virulence genes in long-term evolution, which can cause extensive infection. *Ruminococcus* can produce short-chain fatty acids by degrading cellulose, and short-chain fatty acids are one of the energy sources of intestinal cells. They can maintain the homeostasis of intestinal flora, and carry out immune regulation and anti-inflammatory effects. *Bifidobacterium* can interact closely with intestinal mucosal epithelial cells to form a protective biological barrier, repel pathogenic bacteria through itself and its metabolites, and maintain the balance of intestinal function. *Lactobacillus* can inhibit the reproduction of spoilage bacteria and pathogenic bacteria in the intestine, have immune regulation, promote the production of antibodies, activate macrophages, induce interferon production, and improve the body’s disease-resistance. The imbalance of intestinal flora may lead to low reactive inflammation and produce inflammatory factors to activate microglia and then produce neuroinflammation, resulting in reducing cognitive ability and the occurrence of AD (Caracciolo et al., 2014). Animal experiments show that oral probiotics (*Bifidobacterium lactis*, *Lactobacillus casei*, *Bifidobacterium*, and *Lactobacillus acidophilus*) can significantly improve memory impairment, brain neuron and synaptic damage, glial cell activation, improve the composition of the flora in intestine and brain, reduce the destruction of intestinal barrier and blood-brain barrier, and reduce the level of lipopolysaccharide in plasma and brain, reduce the level of inflammatory factors in the brain and intestine at the level of messenger RNA (Yang et al., 2020). The results showed that the abundance of *Lactobacillus* and *Bifidobacterium* decreased and the abundance of *Escherichia coli* increased in the AD group. Compared with the normal group, the *Ruminococcusi* abundance in the AD group decreased and the fecal cocci abundance increased. It is confirmed again that the bacteria that can cause inflammatory reaction in the intestine of AD patients is higher than those in the normal groups, while the bacteria that inhibit inflammatory reaction were lower.

## Summary

This study found that the diversity of intestinal flora in AD patients is significantly reduced, and the structural changes of intestinal flora significantly increased the abundance of bacteria that may lead to inflammatory response while significantly decreasing the abundance of bacteria that may inhibit the inflammatory response and maintain intestinal function, and increased the level of pro-inflammatory factors. Therefore, we suggest that the occurrence of AD may be related to the low level of the inflammatory response caused by the structural disorder of intestinal flora.

## Advantages and limitations

Many previous studies only studied the changes of intestinal microbiota structure in AD patients or the levels of inflammatory factors in AD patients. Compared with previous correlation studies, the advantage of this study is that we statistically analyzed the structure of gut microbiota between AD patients and controls at both phylum level and genus level. In addition, this study also analyzed the differences in intestinal microbiota structure and inflammatory factor levels in AD patients, and clarified that changes in intestinal microbiota structure could further lead to the occurrence and development of AD by causing low-level inflammatory reaction by combining the two. This study also has some limitations: Firstly, the sample size of this study is relatively small; Secondly, this study did not compare and analyze the differences in gut microbiota structure and inflammatory factor levels among AD patients with different degrees of dementia.

## Data availability statement

The original contributions presented in the study are publicly available. This data can be found here: doi: 10.6084/m9.figshare.21353970.

## Ethics statement

The studies involving human participants were reviewed and approved by the Medical Ethics Committee of the Sixth Medical Center of the PLA General Hospital. The patients/participants provided their written informed consent to participate in this study. Written informed consent was obtained from the individual(s) for the publication of any potentially identifiable images or data included in this article.

## Author contributions

S-SW and X-HL performed the experiments and data analysis and wrote the manuscript. PL designed and supervised the study and edited the manuscript. JL revised statistical analysis. LL critically revised the manuscript. All authors contributed to the article and approved the submitted version.
